# Reappraisal of bone scintigraphy as a new tool for the evaluation of disease activity in patients with rheumatoid arthritis

**DOI:** 10.1038/s41598-021-01104-w

**Published:** 2021-11-08

**Authors:** Sang Jin Lee, Chae Moon Hong, Il Cho, Byeong-Cheol Ahn, Jung Su Eun, Na Ri Kim, Jong Whan Kang, Young Mo Kang

**Affiliations:** 1 Department of Internal Medicine (Rheumatology), School of Medicine, Kyungpook National University Hospital, Kyungpook National University, 130 Dongdeok-ro, Jung-gu, Daegu, 41944 Republic of Korea; 2 Department of Nuclear Medicine, School of Medicine, Kyungpook National University, Daegu, Korea

**Keywords:** Immunology, Rheumatology

## Abstract

We aimed to compare the reliability of bone scintigraphy (BS) and fluorine-18-fluorodeoxyglucose (^18^F-FDG) positron emission tomography (PET)—derived parameters in the detection of active arthritis in 28-joint areas and evaluate the reliability of joint counts between BS and clinical assessment in patients with rheumatoid arthritis (RA). We enrolled 106 patients (67 in the development group and 39 in the validation groups) with active RA who underwent BS, ^18^F-FDG PET/computed tomography (CT), and clinical evaluation of disease activity. We compared the results of BS-derived joint assessment with those of PET-derived and clinical joint assessments. Subsequently we developed a disease activity score (DAS) using BS-positive joints and validated it in an independent group. The number of BS-positive joints in 28-joint areas significantly correlated with the swollen /tender joint counts (SJC/TJC) and PET-derived joint counts. A BS uptake score of 2 (strong positive) was significantly more sensitive compared with a BS uptake score of 1 (weak positive) in detecting a PET-positive joint among the 28-joints. After conducting multivariate analyses including erythrocyte sediment rate (ESR) and patient global assessment (PGA) in addition to BS-derived parameters, BS/DAS was obtained as follows: 0.056 × number of BS-positive joints in 28 joints + 0.012 × ESR + 0.030 × PGA. A significant correlation between BS/DAS and DAS28-ESR was confirmed in the validation group. Strong positive uptake of BS is sensitive and reproducible for the detection of active joints, and can complement the clinical assessment of disease activity in RA.

## Introduction

Rheumatoid arthritis (RA) is a chronic inflammatory joint disorder characterized by the synovial infiltration of active immune cells, which causes the destruction of cartilage, bone, and joint structures^1^. Joint counts performed by experienced physicians are considered crucial in the quantitative assessment of synovitis, which was included in the disease activity score (DAS) 28 for the measurement of RA activity^2^. However, joint counts are limited by an inherent lack of objectivity related to both operator’s factors and patient’s factors^3,4^, thereby increasing the need for more sensitive and reproducible tools to detect synovitis.

Although imaging modalities such as ultrasound (US) and magnetic resonance imaging (MRI) are more sensitive than clinical assessment for detecting joint inflammation^5–7^, it is difficult to assess systemic joint status in patients with RA with these tools^8–11^. Another limitation is the time-consuming nature of these imaging procedures. Recently, fluorine-18-fluorodeoxyglucose (FDG) positron emission tomography (PET)/computed tomography (CT) imaging provided important insights that helped in evaluating disease activity in patients with RA. The FDG PET/CT-derived joint count assessment is a highly reproducible and sensitive tool, and complements the clinical evaluations^12^. However, FDG PET/CT examinations have certain limitations including high levels of radiation exposure, use of expensive core facility, and high costs^13–15^.

Bone scintigraphy (BS), which has long been used in clinical settings for the assessment of inflamed joint distribution, has several advantages for evaluating systemic joints over US, MRI, and FDG PET/CT. BS provides whole-body joint imaging with much less radiation exposure compared with FDG PET/CT^13^ and is a potential tool for quantitative assessment of disease activity in a more affordable and safer way. However, no study has validated the usefulness of BS in the measurement of RA disease activity.

In this study, we aimed to validate the BS-derived quantitative parameters for RA disease activity by comparing BS-derived joint counts with PET-derived joint counts performed in 28-joints. First, BS-derived joint assessment was compared with PET-derived and clinical joint assessments. Subsequently, DAS was developed using BS-positive joints and validated it in an independent group.

## Patients and methods

### Patients and study design

We enrolled 106 patients who had active joints and underwent BS evaluation at Kyungpook National University Hospital from December 2010 to February 2018 in our study. We diagnosed all patients with RA according to the American College of Rheumatology/European League Against Rheumatism criteria of 2010^16^. This study comprised two groups: a development (n = 67) group, in which DAS was derived by both BS and FDG PET/CT, and a validation group (n = 39), in which the DAS was applied. At the time of BS evaluation, we assessed the clinical disease activity including swollen joint count (SJC), tender joint count (TJC), patient global assessment (PGA), erythrocyte sedimentation (ESR), and C-reactive protein (CRP). The clinical assessments of positive joint counts were examined in each patient by the rheumatologists (J.S.E., J.W.K., and N.R.K.) and a BS image analysis was performed by two nuclear medicine physicians (C.M.H. and I.C.). Nuclear medicine physicians were unaware of the clinical positive joint counts and disease activity of the patients. This study was approved by the Institutional Review Board (IRB) and Ethics Committee at Kyungpook National University Hospital (approval number 2018-05-032). The requirement for informed consent was waived by the IRB since the study involved a minimum risk to the enrolled patients and no identifiable information was used. All methods were performed in accordance with the relevant guidelines and regulations.

### FDG PET/CT acquisition protocol and image analysis

A previous study demonstrated the FDG-PET/CT acquisition protocol^12^. All patients fasted more than 6 h, and the blood glucose levels of each patient before the FDG administration was < 150 mg/dL. PET/CT images were obtained from the skull vertex to the feet with the patient in supine position using a Reveal HiREZ 6-slice CT apparatus (CTI Molecular Imaging, Knoxville, TN, USA) 1 h after the intravenous injection of FDG (~ 5 MBq/kg body weight). First, a low-dose CT scan without contrast enhancement was obtained for attenuation correction, and all images were reconstructed using a 3.75-mm slice thickness at 2.5-mm increments. Then a three-dimensional-mode PET scan with a maximum spatial resolution of 6.5 mm was performed for 3 min per bed position. The PET images were reconstructed with a 128 × 128 matrix. When FDG uptake in the joint synovium was higher than normal regional tracer accumulation, the joints were considered positive for active arthritis. PET positive and negative joints were defined as scores 1 and 0, respectively. The volume of interest (VOI) for a PET-positive joint was placed on a joint synovium in PET images, and an iso-contour VOI including all voxels > 42% of the maximum was created; subsequently, the SUVmax value was automatically calculated. The SUVmax was obtained using following formula: maximum activity in the region of interest (MBq/mL) divided by injected dose (MBp)/body weight (g). PET28 was defined as the number of PET-positive joints among the 28-joints. Two experienced nuclear medicine physicians interpreted the PET/CT images and the interpretation of the PET/CT images was repeated 2 months later (by a nuclear medicine physician) or independently (two nuclear medicine physicians).

We previously developed a novel PET/DAS formula using PET/CT after conducting multivariate analyses including ESR and PGA in addition to PET-derived parameters^12^. PET/DAS was derived as 0.063 × PET28 + 0.011 × ESR + 0.030 × PGA.

### BS acquisition protocol and image analysis

BS images were obtained 3–5 h after the injection of 740 MBq (20 mCi) of Tc-99m hydroxymethylene diphosphonate (HDP). A dual-headed gamma camera equipped with a high-resolution collimator (Infinia, GE, Milwaukee, WI, USA) was used to obtain anterior and posterior whole-body images, along with the static images of hands. The regions of interest (ROIs) were drawn around the 28-joints and a semi-quantitative analysis was performed in each joint and the results were compared with the reference backgrounds. For large joints including wrist joints and small joints, the left side of skull in the whole body image and right radius in the static image of the hand were used as a reference background, respectively. Then, the 28-joints of each patient were scored as 0–2 (0: negative, 1: weak positive, 2: strong positive; Supplementary Fig. S1). The visual interpretation of BS images was performed by two nuclear medicine physicians (C.M.H. and I.C.). The interpretation of the BS images was repeated 2 months later (C.M.H.) or independently (C.M.H. and I.C.). BSS28 was defined as the number of BS-positive joints among the 28-joints.

### Statistical analysis

The baseline clinical data were expressed as means ± SD for continuous variables or as numbers and percentages for categorical variables. To compare BS and PET/CT in terms of the detection of active joints, the significant differences between variables were calculated using the chi-square test and Mann-Whitney test. The correlations between the BS-derived parameters and other disease activity measures were calculated using the Pearson’s correlation test, with Bonferroni’s correction. The intra-observer (the nuclear medicine physician, 2 month intervals) and inter-observer (between the two nuclear medicine physicians or between a nuclear medicine physician and rheumatologists) in the 28-joints counts were calculated using the Cohen κ-test and intraclass correlation coefficient (ICC). A kappa value of 0–0.20 was considered poor, 0.21–0.40 as fair, 0.41–0.60 as moderate, 0.61–0.80 as good, and 0.81–1.00 as excellent^17,18^. ICCs between the BSS28 and PET28, TJC28 in the development group were calculated using a two-way mixed-effects model and the Bland–Altman approach^19^.

For the development of DAS using BS, univariate and multivariate analyses were conducted using the linear regression model to evaluate the association among clinical factors, including BS-derived parameters, and disease activity measures in patients with RA. After the generation of BS/DAS, we calculated it for each patient in the validation group (n = 39). Pearson’s correlation test was utilized to compare the correlation between BS/DAS and DAS28-ESR. *P *values < 0.05 were considered significant. All statistical analyses were performed using SPSS version 19 software (IBM, Chicago, IL, USA) and GraphPad Prism version 5 (GraphPad, San Diego, CA, USA) was used to generate the graphics.

### Ethics approval and consent to participate

This study was approved by the Institutional Review Board at confirmed Kyungpook National University Hospital (2018-05-032).

## Results

### Baseline characteristics in the development and validation groups

We enrolled 86 patients with active RA in the development group (n = 67) and validation group (n = 39) who underwent BS, disease activity evaluation, and/or FDG-PET/CT at the same time. The mean ages of the development and validation groups at the time of disease evaluation were 68 and 67 years, respectively. The proportion of women was similar between the two groups. Additionally, the mean DAS28-ESR of the development and validation groups were 6.81 and 6.43, respectively, with all patients in both groups showing moderate to high disease activity. In both groups, 53 patients (79.1%) and 28 patients (71.8%) were naïve to disease-modifying antirheumatic drugs (DMARDs) (Table 1).

**Table 1:** Baseline characteristics of the study participants with rheumatoid arthritis

	Development group (n = 67)	Validation group (n = 39)
Age (years) at bone scan/PET	67.69 ± 12.8	66.64 ± 12.9
Age (years) at diagnosis	65.78 ± 14.8	65.24 ± 13.0
Sex, female	43 (64.2)	26 (66.7)
RF (IU/ml)	124.37 ± 238.0	121.72 ± 259.0
Anti CCP Ab (U/ml)	180.96 ± 209.8	124.48 ± 193.7
Seropositive	44 (65.7)	16 (41.0)
ESR (mm/h)	64.13 ± 31.2	67.38 ± 26.9
CRP (mg/dl)	8.15 ± 5.8	7.21 ± 6.1
DAS28-ESR	6.81 ± 1.1	6.43 ± 1.1
DAS28-CRP	6.41 ± 1.2	5.42 ± 1.1
PGA	71.82 ± 17.9	66.79 ± 16.8
DMARD-naïve patients	53 (79.1)	28 (71.8)
Active joint count		
Swollen joints (28)	12.43 ± 7.4	9.79 ± 6.9
Tender joints (28)	14.10 ± 7.1	11.69 ± 7.2

Data are expressed as means ± SD for continuous variables or numbers and percentages for categorical variables. RF, rheumatoid factor; *antiCCP* anti-cyclic citrullinated peptide, *ESR* erythrocyte sedimentation rate, *CRP* C-reactive protein, *DAS* disease activity score, *PGA* patient global assessment, *DMARD* disease-modifying antirheumatic drugs

### Correlations between BS-derived parameters and other disease activity measures in the development group

**Figure 1:**
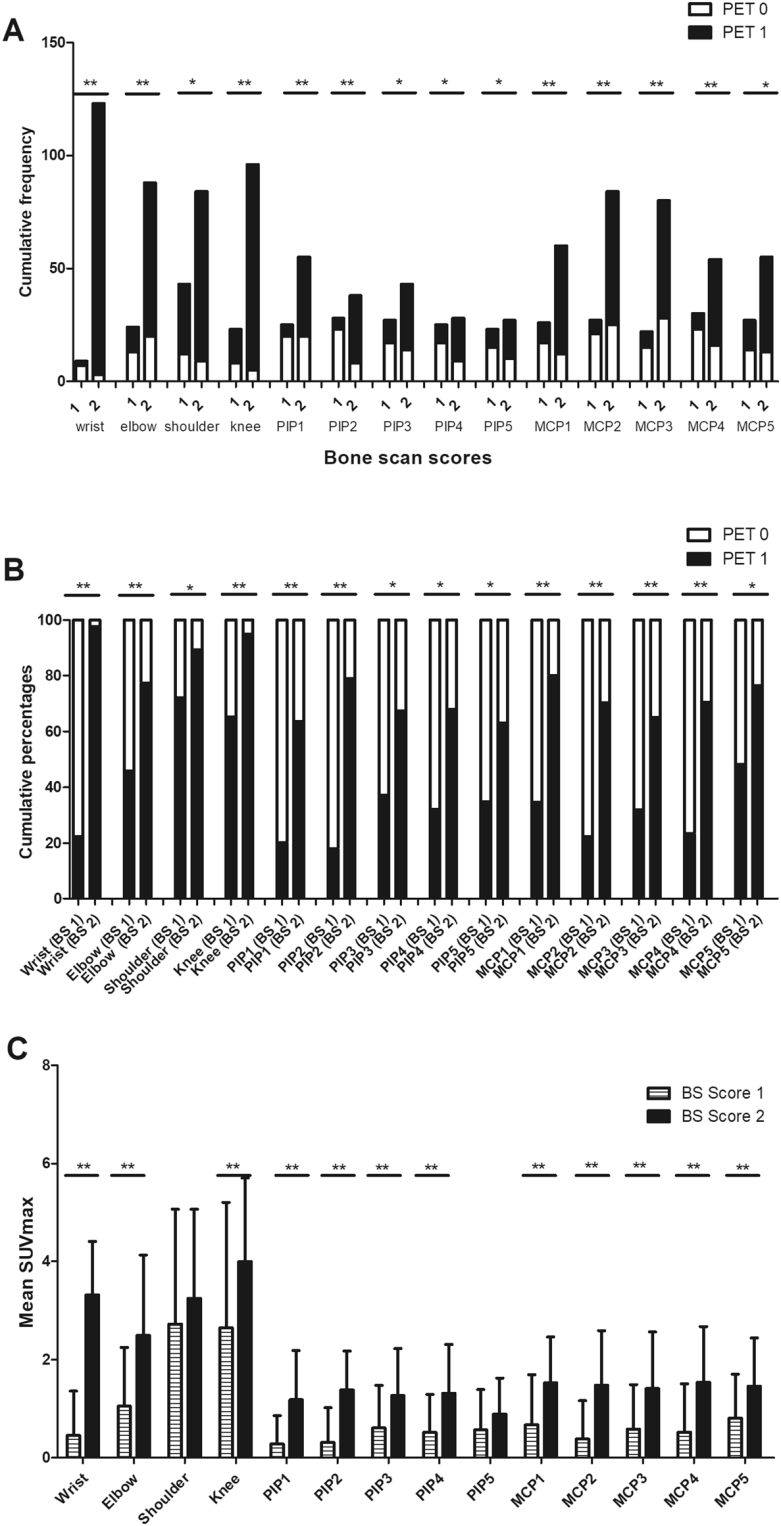
Comparison of positron emission tomography (PET) and bone scintigraphy (BS) in the detection of active joints. The frequencies (**A**), percentages (**B**) and the mean SUVmax (**C**) of PET positive joints were expressed according to BS scores in the affected individual joints among the 67 patients who underwent PET and BS. A total of 134 frequencies were observed for each joint. In total, 12 frequencies in knees were excluded in the analysis because those indicate the status of total knee replacement arthroplasty. When FDG uptake in the joint synovium was higher than normal regional tracer accumulation, the joints were considered positive for active arthritis. PET positive and negative joints were defined as scores 1 and 0, respectively. The 28-joints of each patient in BS were scored as 0–2 (0: negative, 1: weak positive, 2: strong positive). The BS uptake score of 2 was used as a criterion for diagnosing a BS positive joint. MCP, metacarpophalangeal joint; PIP, proximal interphalangeal joint; SUV, standardized uptake values. **p < 0.005; *p < 0.05

**Figure 2:**
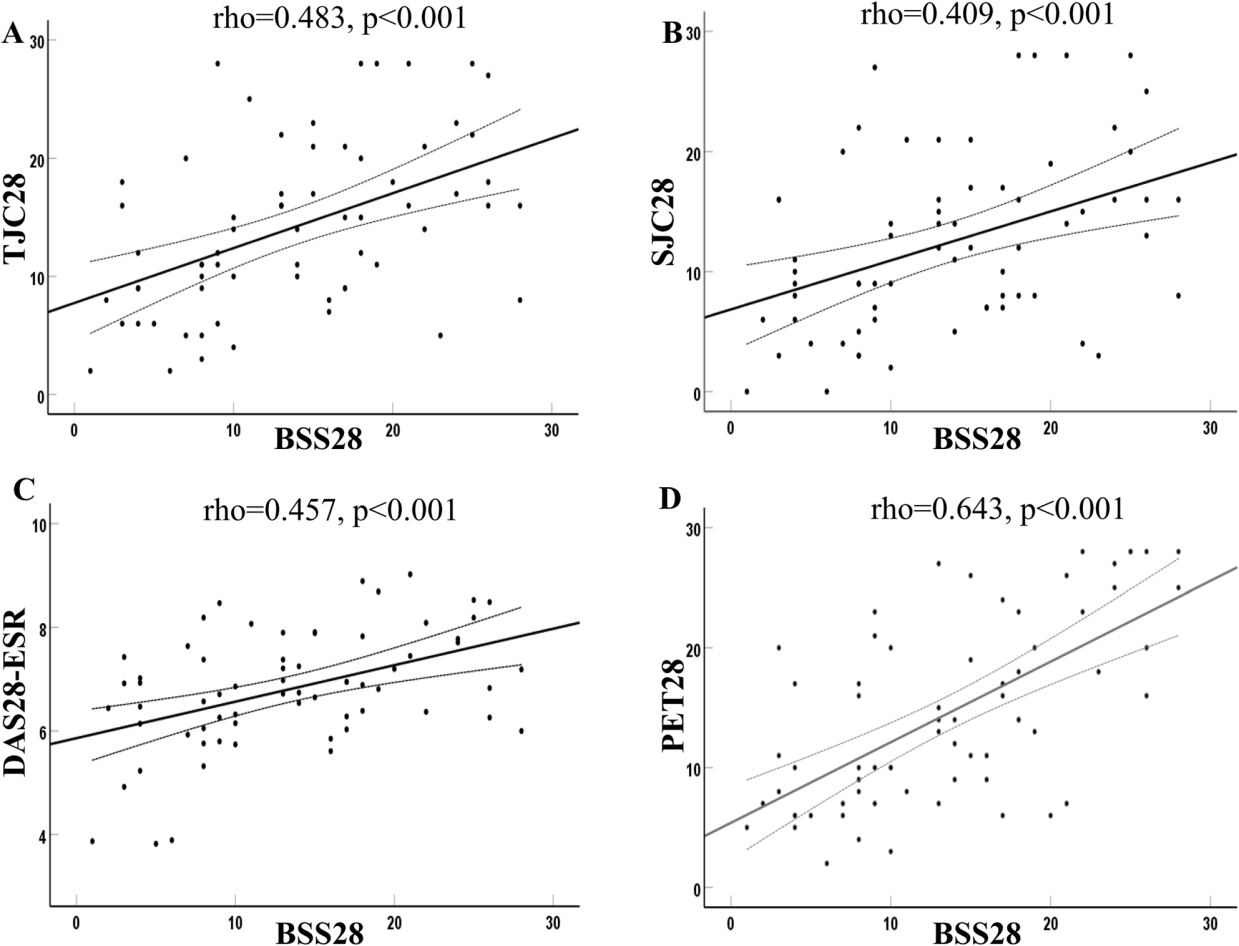
Correlation between positive findings in the joints assessed by PET, BS, and clinical assessment. The number of bone scintigraphy positive joints in 28-joints (BSS28) was significantly correlated with the tender joint counts in 28 joints (TJC28) (r = 0.483, *p* < 0.001) (**A**), the swollen joint counts in 28 joints (SJC28) (r = 0.409, *p* = 0.001) (**B**), DAS28-ESR (r = 0.457, *p* < 0.001) (**C**), and the number of PET-positive joints in 28 joints (PET28) (r = 0.643, *p* < 0.001) (**D**). Each symbol represents an individual data point, and the dotted lines represent the 95% confidence interval. The correlation coefficients and *p* values were calculated using the Pearson’s correlation test. ESR, erythrocyte sedimentation rate.

To compare the reliability of BS and PET/CT in the detection of active joints, the individual affected joints examined by BS, in terms of cumulative frequencies and percentages of involvement (Fig. 1A, B) of the individual joint, were expressed based on the positive joint counts and SUVmax on PET/CT (Fig. 1C). A BS uptake score of 2 was significantly more sensitive compared with a BS uptake score of 1 in detecting a PET-positive joint among the 28-joints (Fig. 1A, B). Thus we used the BS uptake score of 2 as a criterion for diagnosing a BS positive joint. At the time of BS and PET/CT evaluation, the clinical disease activity was assessed using SJC28, TJC28 and DAS28-ESR. To investigate the correlation between BSS28 and clinical disease activity, the BSS28 was compared with the clinical parameters including TJC28, SJC28 and DAS28-ESR. The BSS28 was significantly correlated with TJC28 (r = 0.483, *p* < 0.001), SJC 28 (r = 0.409, *p* = 0.001), and DAS28-ESR (r = 0.457, *p* < 0.001) (Fig. 2A–C). The BSS28 was also significantly correlated with PET28 (r = 0.643, *p* < 0.001) (Fig. 2D).

### Reliability of joint counts between BSS28 and other disease activity measures in the development group

Kappa values between BS results and clinical assessments of the individual joints ranged from 0.033 to 0.457. However, these values indicated constant fair to moderate agreement, except for the knee and shoulder joints. The reliability values between BSS28 and SJC28/TJC28 assessed by ICCs at the patient level in 28-joints were 0.585 (95% confidence interval: 0.324–0.745) and 0.646 (0.424–0.783), respectively (Table 2). The reliability between BS and PET/CT for joint counts ranged from 0.194 to 0.703 by kappa values at the individual joint and 0.782 (0.646–0.866) by ICCs at the patient level in 28-joints, respectively. These reliability values were higher than those between BS results and clinical assessments (Table 2).

**Table 2:** Reliability between bone scintigraphy and other disease activity measures for joint counts

	Inter-observer reliability (SJC)		Inter-observer reliability (TJC)		Inter-observer reliability (PET)	
	Ƙ	P-value	ƙ	P-value	ƙ	P-value
PIP_1	0.249	0.002	0.217	0.005	0.370	< 0.001
PIP_2	0.457	< 0.001	0.454	< 0.001	0.586	< 0.001
PIP_3	0.277	< 0.001	0.317	< 0.001	0.374	< 0.001
PIP_4	0.256	< 0.001	0.297	< 0.001	0.469	< 0.001
PIP_5	0.212	0.004	0.259	0.001	0.305	< 0.001
MCP_1	0.401	< 0.001	0.410	< 0.001	0.580	< 0.001
MCP_2	0.293	< 0.001	0.274	0.001	0.524	< 0.001
MCP_3	0.273	0.001	0.260	0.002	0.441	< 0.001
MCP_4	0.300	0.001	0.300	0.001	0.560	< 0.001
MCP_5	0.243	0.005	0.244	0.004	0.501	< 0.001
Wrist	0.259	0.001	0.273	< 0.001	0.703	< 0.001
Elbow	0.240	0.005	0.187	0.022	0.454	< 0.001
Shoulder	0.103	0.193	0.033	0.676	0.194	0.010
Knee	0.248	0.005	0.181	0.037	0.256	0.001

Inter-observer reliability was calculated using the Cohen κ-test and intraclass correlation coefficients (ICC). For the SJC and the TJC, the ICC was 0.585 (95% CI 0.324–0.745) and 0.646 (95% CI 0.424–0.783), respectively. For the PET, the ICC was 0.782 (95% CI 0.646–0.866). *SJC* swollen joint counts, *TJC* tender joint counts, *PIP* proximal interphalangeal joint, *MCP* metacarpophalangeal joint, *CI* confidence intervals

**Figure 3:**
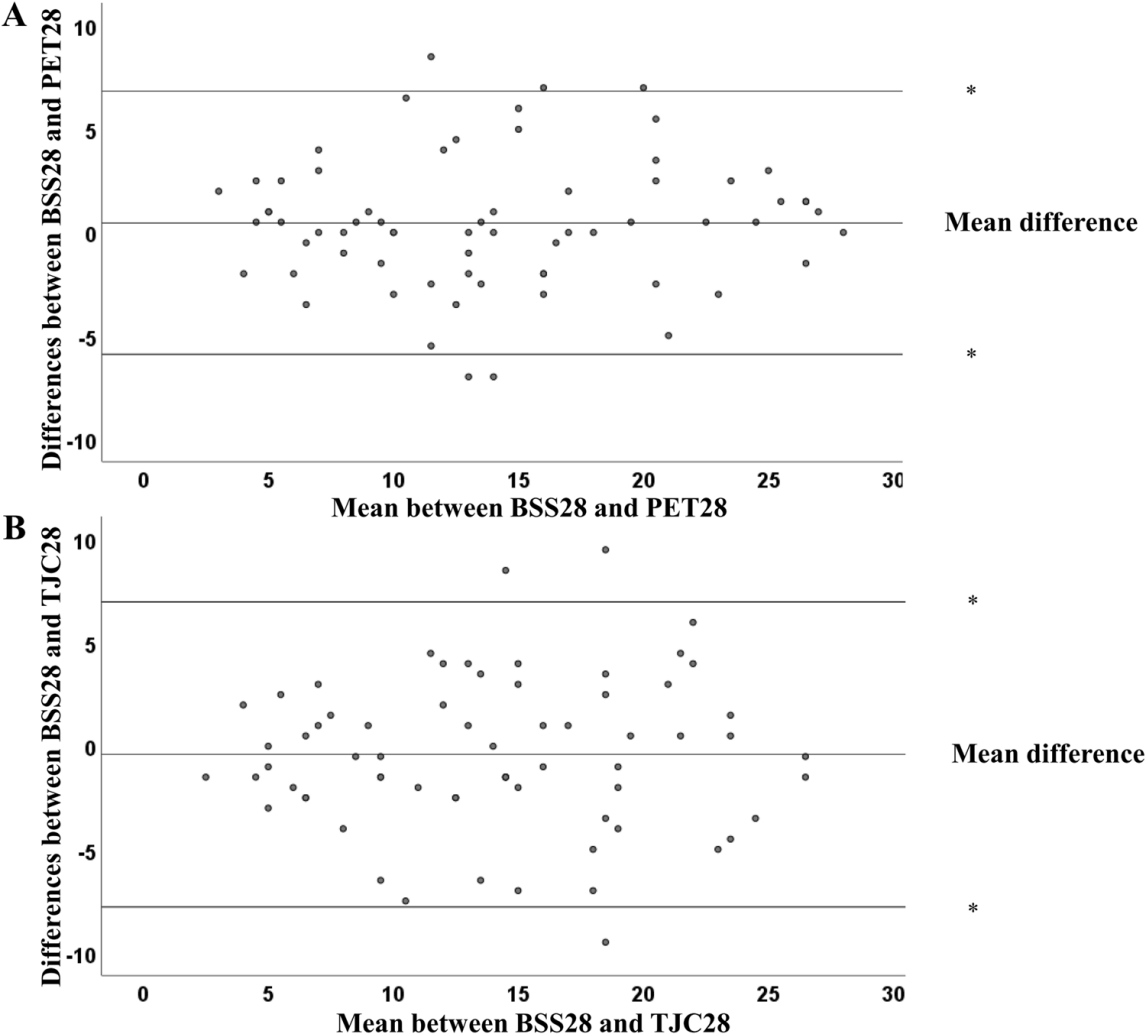
Bland–Altman plot showing the interobserver reliability of assessment of the number of bone scintigraphy-positive joints in 28 joints (BSS28) in relation to PET positive joint count in 28-joints (PET28) (**A**) and the tender joint count in 28-joints (TJC28) (**B**). Mean between BSS28 and PET28 = average of the joint count as determined by the BSS28 and the joint count as determined by the TJC28. The mean differences between the BSS28 and PET28, and the BSS28 and TJC 28 were 0.46 and − 0.40, respectively, and the majority of plots (62 of 67 [92.5%] and 64 of 67 [95.5], respectively) were within the upper and lower limits of 2 SD (lines with asterisks).

The level of reliability of the BSS28 in relation to the PET28 and TJC28 was further illustrated by the Bland–Altman plots. The mean differences between the BSS28 and PET28/TJC 28 were 0.46 and − 0.40, respectively. The majority of plots (62 of 67 (92.5%) and 64 of 67 (95.5%), respectively) were within the upper and lower limits of 2 SD (Fig. 3A, B).

When the intra-observer reliability of the nuclear medicine physician was evaluated, the kappa values at the individual joint showed moderate to excellent agreement, and the ICC values at the patient level showed an excellent reliability (0.938, 0.840–0.976). Furthermore, the ICC values of the inter-observer results (between two nuclear medicine physicians) were good in the 28-joints counts (0.830, 0.560–0.935) (Supplementary Table S1).

### Development of the DAS using BS (BS/DAS)

For the development of BS/DAS, a linear regression model was used to analyze the BSS28 and DAS28-ESR. After conducting multivariate analyses including ESR or CRP, and PGA in addition to BSS28, the values of ESR or CRP, PGA, and BSS28 were independently associated with the DAS28-ESR/CRP (Supplementary Table S2). Using these parameters, the BS/DAS was derived based on the regression coefficients as the following formula:

$$ [BS/DAS = 0.056 \times BSS28 + 0.012 \times ESR + 0.030 \times PGA] $$

### Validation of the BS/DAS in the independent group

**Figure 4:**
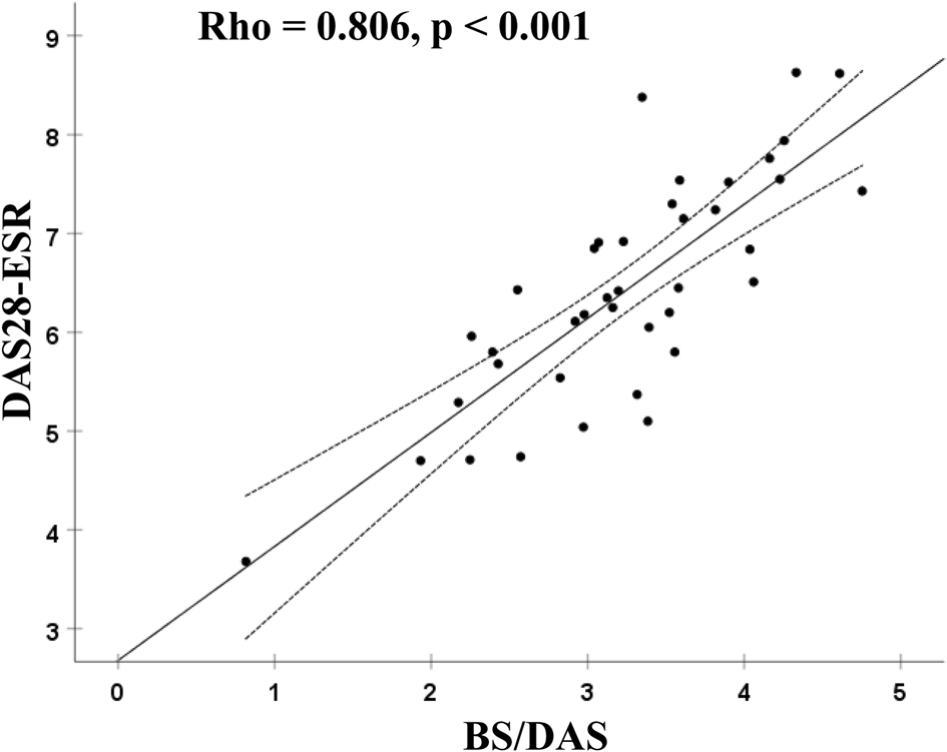
The correlation between BS/DAS and DAS28-ESR. BS/DAS (n = 39) was significantly correlated with DAS28-ESR (r = 0.806, *p* < 0.001). The correlation coefficient and *p*-value were derived by conducting the Pearson’s correlation test. *BS* bone scintigraphy, *DAS* disease activity score, *ESR* erythrocyte sedimentation rate.

In the validation group, 28 patients were naïve to DMARDs, with 11 showing inadequate responses. Disease activities such as DAS28-ESR/CRP in the validation group were not significantly different from the development group (Table 1). The BS/DAS in the validation group were significantly correlated with DAS28-ESR (r = 0.806, *p* < 0.001) (Fig. 4). BS/DAS were also significantly correlated with the DAS28-CRP, TJC28, and SJC28 (Supplementary Table S3).

## Discussion

This study had two main results. First, the BS-derived joint assessment significantly correlated with clinical and PET/CT-derived joint counts, and its reliability was good for both clinical and PET/CT-derived findings. Second, we developed the disease activity formula, the BS/DAS, which is composed of the BSS28, levels of ESR, and the PGA. Additionally, the formula was confirmed in a validation group.

In our previous study, FDG-PET/CT could serve as a sensitive and reproducible method for assessing disease activity in patients with RA^12^. Although the radiation dose is reduced with more advanced scanners, an increase in radiation exposure is one of a major safety concern in this procedure^15^. In Korea, the average radiation doses of PET/CT and BS are 12.2 and 4.2 mSv, respectively, as estimated by a national survey^14,15^. Furthermore, the cost of conducting a PET/CT examination is high and this procedure required the use of accompanying facilities including the tracer production, so PET/CT study may not be possible in small to moderate sized facilities.

Therefore, the use of FDG PET/CT for evaluating disease activity in a routine clinical practice remains challenging. On the contrary, BS imaging for active joint count has much less radiation exposure than PET/CT imaging, while it provides similar reliable results in patients with RA. The correlation coefficient of a BS/DAS formula for representing DAS28-ESR in each patient in the validation group in this study is comparable to that of PET/DAS formula in a previous study (r = 0.806, *p* < 0.001 vs r = 0.843, *p* < 0.001, respectively)^12^.

BS is a highly sensitive diagnostic technique of nuclear imaging that uses a radiotracer to evaluate the distribution of active bone formation^20^. Solid tumors with high affinity for bone, metabolic bone diseases, and joint diseases such as chronic inflammatory arthritis and osteoarthritis (OA) are indications for BS evaluation^20^. BS has been used for the differential diagnosis of RA, OA, spondyloarthritis, and unclassified arthritis in the field of rheumatology^21–23^. Additionally our results show that joint count by BS evaluation is a reproducible method for assessing bone changes in the affected synovitis, with good reliability between observers, thus BS can be used for measuring disease activity in patients with RA. Therefore, this tool may help physicians worldwide who take care of RA patients, but without well-trained expertise or expensive facilities such as PET/CT. Moreover, as an objective tool for identifying arthritis in our study, BS may be applied for the differential diagnostic process of unclassified arthritis, which is important to estimate affected joints and their location. However, follow-up study is needed to clarify whether changes of BS uptake in patient with RA improve or remain after treatment.

Although previous studies on disease activity assessment using BS in patients with RA were limited, two reports showed a significant correlation between the regional uptake for large joints on BS and disease activity^24,25^. These studies did not evaluate 28-joints including small joints and did not compare the BS values with DAS28. According to the analysis of the affected joint in a large cohort with RA patients, tender joints were frequently observed in large joints, while swollen joints were frequently observed in the small joints of the hands^26^. Thus, evaluating large joints alone is not sufficient to represent the accurate disease activity. Furthermore, the reliability of BS for clinical assessment of large joints such as knee and shoulder joints was relatively lower than that of other joints in our study. Therefore, joint count based on the BS values of 28-joint areas including both small and large joints should provide a more objective parameter for disease activity assessment. Because it is important to determine the cut off value for BS score to assess for synovitis in patients with RA, we compared affected individual joints between BS scores and PET/CT examination. A BS uptake score of 2 was significantly more reliable than a BS uptake score of 1 in detecting PET-positive joint at 28 joints. Thus we used the BS uptake score of 2 as a criterion for BS positive joint.

Despite the crucial role of RA disease activity measurement in detecting synovitis, clinical assessments of joint counts are not routinely performed in clinics because reliability of joint count assessments, considering both the intra-observer and inter-observer variabilities, needs to be explored further^27^. The intra-observer reliability of ICCs for the clinical assessment of joint counts by healthcare professionals ranged from 0.47 to 0.98 in both TJC and SJC^28^, whereas the reliability of kappa value at the joint level varied from fair to good in SJC^29^, thereby suggesting the inconsistent joint assessment in clinical practice. Furthermore, the range of inter-observer reliability assessed with the ICCs and the kappa value was dependent on the variation among study samples in finding a positive joint count (from 0.29 to 0.98, from poor to excellent, respectively)^30,31^. By contrast, joint counts by BS evaluation are a reproducible method for assessing synovitis, with excellent inter-observer and intra-observer reliability.

Surprisingly, when observing the ICC values of reliability between BS and PET/CT findings in 28-joints, the ICC between BSS28 and PET28 was 0.782 (0.646–0.866). Furthermore, the ICC values between BS28 and TJC28 were comparable to those between PET28 and TJC28 (0.646 and 0.728, respectively)^12^, implicating that the BSS28 and clinical assessments that were performed by experienced clinicians had a good reliability. We also developed a novel BS/DAS formula derived from the results of BS assessment alone, without using the results of joint assessment performed by experienced clinicians. This formula was confirmed in an independent validation group of RA patients. The BS/DAS, which may overcome the variability of clinical evaluation by joint assessors with diverse backgrounds, can complement the use of the DAS28-ESR and may provide similar results compared with more advanced modality such as PET/CT for evaluation of disease activity.

It was reported that US and MRI have the excellent capability in the evaluation of inflammatory arthritis, and their scores significantly correlated with DAS 28, proving their utility in the diagnosis and monitoring of patients with RA^8,32,33^. One study demonstrated that there was a powerful linear relationship between scores from MRI and PET/CT in the evaluation of arthritis, despite the fact that these modalities have different ways to identify synovitis^7^. Because FDG-PET/CT is considered as an excellent tool for evaluating inflammatory reaction in the joints, we assessed the utility of the BS in patients with RA. Given that the use of PET/CT in daily practice is challenging, this work can be a initial step (transversal study) before a clinical trial using BS to monitor the disease activity in patients with RA. Although US is easily accessible and has advantage of real-time examination and MRI has advantage of visualizing intra-osseous abnormality, they are time-consuming and have definite limitations in the evaluation of systemic joints^33^. On the contrary, PET/CT and BS images show the involvement of the whole joint pattern for synovial inflammation^12,20^. Especially, given the relative low cost and widespread availability of BS in an era of more advanced imaging tools, our findings could provide new insight into the BS evaluation in patients with RA.

There are two limitations in this study. First, BS reflects bone remodeling and uptakes in knee joints can be observed in patients with knee OA^21^, regardless of RA disease activity. Second, patients were enrolled at a single center, thus multicenter studies of BS validation are warranted to determine whether our findings are generalizable.

## Conclusion

In conclusion, BS is a sensitive and reproducible method for the detection of active joints, and can complement the clinical assessment of disease activity in RA. Despite the availability of more advanced imaging modality such as PET/CT, considering their costs, and the radiation and sensitivity for evaluating active joints, BS may still be comparable to this advanced imaging method in terms of assessing disease activity in patients with RA. In the future, the incorporation of deep learning from BS images into computer-aided evaluation is promising for the assessment of disease activity in patient with RA.

## Supplementary Information

Supplementary Information.

## Abbreviations

RA: Rheumatoid arthritis
DAS: Disease activity score
US: Ultrasound
MRI: Magnetic resonance imaging
FDG: Fluorodeoxyglucose
PET: Positron emission tomography
CT: Computed tomography
BS: Bone scintigraphy
SJC: Swollen joint count
TJC: Tender joint count
PGA: Patient global assessment
ESR: Erythrocyte sedimentation
CRP: C-reactive protein
VOI: Volume of interest
HDP: Hydroxymethylene diphosphonate
ROI: Regions of interest
ICC: Intraclass correlation coefficient
DMARDs: Disease-modifying antirheumatic drugs
OA: osteoarthritis
