# Supplementary material for: Reappraisal of bone scintigraphy as a new tool for the evaluation of disease activity in patients with rheumatoid arthritis
Source: Sci Rep. 2021 Nov 8;11:21809. doi: 10.1038/s41598-021-01104-w (PMC8575878; doi:10.1038/s41598-021-01104-w)
Supplement: Supplementary file 1 — Supplementary Information. [file 41598_2021_1104_MOESM1_ESM.docx]

Supplementary table 1. Reliability between nuclear medicine physicians for joint counts

|  | **Intra-observer reliability** | | | | | **Inter-observer reliability** | | | | |
| --- | --- | --- | --- | --- | --- | --- | --- | --- | --- | --- |
|  | **ƙ** | **P-value** | | **ICC** | **P-value** | **ƙ** | **P-value** | | **ICC** | **P-value** |
| PIP_1 | 0.501 | 0.001 | |  |  | 0.139 | 0.198 | |  |  |
| PIP_2 | 0.580 | <0.001 | |  |  | 0.377 | 0.003 | |  |  |
| PIP_3 | 0.526 | <0.001 | |  |  | 0.316 | 0.017 | |  |  |
| PIP_4 | 0.678 | <0.001 | |  |  | 0.283 | 0.012 | |  |  |
| PIP_5 | 0.689 | <0.001 | |  |  | 0.496 | <0.001 | |  |  |
| MCP_1 | 0.741 | <0.001 | |  |  | 0.588 | <0.001 | |  |  |
| MCP_2 | 0.598 | <0.001 | |  |  | 0.337 | 0.006 | |  |  |
| MCP_3 | 0.718 | <0.001 | |  |  | 0.395 | 0.005 | |  |  |
| MCP_4 | 0.740 | <0.001 | |  |  | 0.537 | <0.001 | |  |  |
| MCP_5 | 0.596 | <0.001 | |  |  | 0.368 | 0.003 | |  |  |
| Wrist | 0.655 | <0.001 | |  |  | 0.065 | 0.260 | |  |  |
| Elbow | 0.938 | <0.001 | |  |  | 0.142 | 0.088 | |  |  |
| Shoulder | 0.632 | <0.001 | |  |  | 0 | ? | |  |  |
| Knee | 0.678 | <0.001 | |  |  | 0.174 | 0.057 | |  |  |
| 28 joints |  |  | 0.938 (CI:0.840-0.976) | | <0.001 |  |  | 0.830 (CI:0.560-0.935) | | <0.001 |

Intra-observer and inter-observer reliabilities were calculated using the Cohen κ-test and intraclass correlation coefficients (ICC). PIP, proximal interphalangeal joint; MCP, metacarpophalangeal joint; CI, confidence intervals.

Supplementary table 2. Association of DAS28-ESR/DAS28-CRP with bone scintigraphy-derived parameters via univariate and multivariate linear regression

| **Variate** | **Univariate analysis** | | **Multivariate analysis** | |
| --- | --- | --- | --- | --- |
|  | β ± SE | P-value | β ± SE | P-value |
| **BSS28** | 0.070 ± 0.017 | <0.001 | 0.056 ± 0.014 | <0.001 |
| **ESR (mm/h)** | 0.014 ± 0.004 | 0.001 | 0.012 ± 0.003 | <0.001 |
| **PGA** | 0.034 ± 0.007 | <0.001 | 0.030 ± 0.006 | <0.001 |
|  |  |  |  |  |
| **BSS28** | 0.071 ± 0.018 | <0.001 | 0.053 ± 0.014 | <0.001 |
| **CRP (mg/l)** | 0.105 ± 0.022 | <0.001 | 0.055 ± 0.018 | 0.004 |
| **PGA** | 0.041 ± 0.006 | <0.001 | 0.031 ± 0.006 | <0.001 |

BSS28, the number of bone scintigraphy-positive joints in 28-joints; ESR, erythrocyte sedimentation rate; PGA, patient’s global assessment; CRP, c-reactive protein.

Supplementary Table 3. Correlation between Bone scan/DAS and disease activity in the validation group.

|  |  | BS/DAS | DAS28-ESR |
| --- | --- | --- | --- |
| All (n = 39) |  |  |  |
| DAS28-CRP | Rho | 0.740 | 0.916 |
|  | P-value | <0.001* | <0.001* |
| TJC28 | Rho | 0.561 | 0.876 |
|  | P-value | <0.001* | <0.001* |
| SJC28 | Rho | 0.600 | 0.912 |
|  | P-value | <0.001* | <0.001* |

The correlation between the BS/DAS and disease activity was calculated using Pearson’s correlation test.

BS, bone scintigraphy, DAS, disease activity score; ESR, erythrocyte sedimentation rate; CRP, C reactive protein; TJC28, tender joint counts in 28 joints; SJC28, swollen joint counts in 28 joints.

* indicates a significant correlation.

Supplementary figure 1.

Bone scintigraphy images were obtained anterior and posterior whole-body images, along with the static images of hands. Regions of interest (ROIs) were drawn around the 28-joints and semi-quantitative analysis was performed in each joint by reference backgrounds. For large joints including wrist joints, left side of skull in whole body scan image was used as a reference background (A). A right radius in static image of hand were used as a reference background (B). Then, the 28-joints of the each patient were scored as 0–2 (0: negative, 1: weak positive, 2: strong positive). The Left shoulder and elbow were scored as 0 and right shoulder, knee and left wrist were scored as 1 and right wrist, elbow and left knee were scored as 2 (A). The right 1st MCP, 2nd-5th PIP joints and left 4th, 5th PIP joints were scored as 1 and other joints except formers were scored as 2 in small joints (B). PIP, proximal interphalangeal joint; MCP, metacarpophalangeal joint.


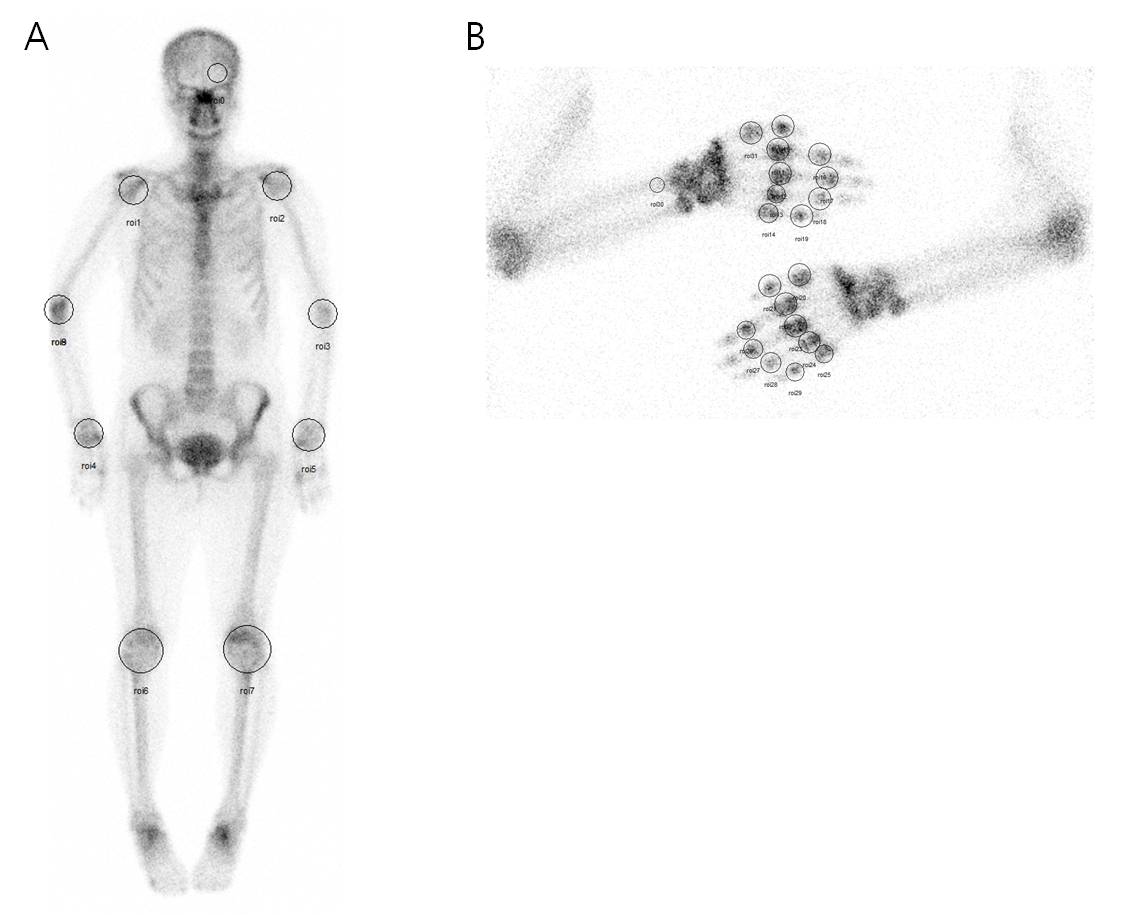


**Reappraisal of bone scintigraphy as a new tool for the evaluation of disease activity in patients with rheumatoid arthritis**

Sang Jin Lee^1^*, Chae Moon Hong^2^*, Il Cho^2^, Byeong-Cheol Ahn^2^, Jung Su Eun^1^, Na Ri Kim^1^, Jong Whan Kang^1^, Young Mo Kang^1^

^1^Department of Internal Medicine, School of Medicine, Kyungpook National University, Korea, ^2^Department of Nuclear Medicine, School of Medicine, Kyungpook National University, Korea.

*These authors contributed equally to this work.

**Corresponding author**: Young Mo Kang, M.D., Ph.D.

Department of Internal Medicine (Rheumatology), Kyungpook National University Hospital

130 Dongdeok-ro, Jung-gu, Daegu 41944, Republic of Korea

Phone: +82-53-420-5495, Fax: +82-53-426-2046, E-mail: ymkang@knu.ac.kr

**Funding:** This work was supported by the National Research Foundation of Korea Grants funded by the Korean Government (NRF-2019R1F1A1062038 and 2017R1A2B2008288) and was supported by Biomedical Research Institute grant, Kyungpook National University Hospital (2017)

**Word count: 3418**
